# The Effect of Yellow Filter Use on Standard Automated Perimetry and Contrast Sensitivity in Healthy Individuals

**DOI:** 10.7759/cureus.51912

**Published:** 2024-01-08

**Authors:** George I Triantafyllopoulos, Costas H Karabatsas, Evangelos Pateras, Aristeidis Chandrinos, Dimitrios Kapralos, Iordanis Georgiou, Anastasia Tsiogka, Dimitrios Kourkoutas

**Affiliations:** 1 Department of Biomedical Sciences, Faculty of Health Sciences, University of West Attica, Athens, GRC; 2 Department of Ophthalmology, 401 General Military Hospital of Athens, Athens, GRC; 3 Department of Ophthalmology, General Hospital of Athens "G. Gennimatas", Athens, GRC

**Keywords:** yellow filters, standard automated perimetry, visual fields, pelli-robson, contrast sensitivity, healthy individuals

## Abstract

Purpose

The purpose of this study is to investigate the effect of two yellow filters (category 1: visible light transmission {VLT} from 80% to 43%) of Essilor (Kiros® and Lumior®) on standard automated perimetry (SAP) indices and Pelli-Robson (PR) contrast sensitivity (CS) testing in healthy individuals.

Materials and methods

This study is a prospective comparative study of 31 eyes of 31 healthy individuals aged 32.14 (8.13) years (14 males and 17 females). All participants underwent a series of three visual field (VF) examinations (24-2, Swedish Interactive Thresholding Algorithm {SITA} standard) with the Humphrey field analyzer (HFA II 740, Carl Zeiss Meditec, Jena, Germany) and three CS examinations with the PR chart (Precision Vision, Inc., Woodstock, IL). VF and CS examinations were carried out as follows: (a) no filter (NF), (b) with the yellow filter Kiros® (KIROS), and (c) with the yellow-orange filter Lumior® (LUMIOR). The effect of the two yellow filters on global VF indices (glaucoma hemifield test {GHT}, mean deviation {MD}, pattern standard deviation {PSD}, and visual field index {VFI}) and on CS score was evaluated and compared.

Results

When comparing the three pairs NF-KIROS, NF-LUMIOR, and KIROS-LUMIOR, no difference was presented on the global VF indices. However, a statistically significant difference was detected in the CS scores for all three pairs, favoring KIROS. It is important to note that while this difference was statistically significant, it did not reach clinical significance.

Conclusions

The use of yellow filters (category 1: VLT of 75% and 65%) does not affect the global VF indices and the CS of healthy individuals but significantly improves their CS score. Further studies are required to explore the clinical significance of these findings.

## Introduction

The effect of yellow tint filters has been extensively studied by several researchers on many aspects of visual function. A literature review by Clark [1] in 1969 concluded that the visual acuity of normal individuals through yellow-, brown-, or orange-colored filters was identical to visual acuity through a neutral tint filter.

The effect of yellow filters (ranging from ultraviolet {UV} cutoff of 400 nm to 550 nm and visible light transmission {VLT} from 50% to 90%) has been previously investigated in individuals with normal visual function. Numerous parameters have been investigated in these studies, including contrast sensitivity (CS) [2-8], visual acuity [2,4,5], visual evoked potentials [4], color perception [5,9], pedestrian detection [10], temporal response to low-contrast targets and traffic hazards [11], adaptation-convergence function [5], luminance [12], pupillary mydriasis [13], standard automated perimetry (SAP) [14], reading speed [15], athletic activities [16], daily visual function [17], and visual object categorization in normal aging [18].

Lenses with a yellow tint (cutoff: 450 nm) are known to enhance the contrast sensitivity of bright objects against a blue-based background. This enhancement results from the selective reduction of short-wavelength light by the yellow lenses, contributing to improved contrast of overlying objects [5]. Recent advancements in contrast-enhancing filters, such as Kiros® and Lumior®, are currently recommended to enhance contrast perception and improve vision and visual comfort, as per the claims of the manufacturers. However, findings from a recent Cochrane systematic review [19] indicated that blue-light-filtering lenses did not outperform non-blue-light-filtering lenses in reducing eye strain associated with computer use. Moreover, these lenses had little to no effect on best-corrected visual acuity (BCVA), and their impact on sleep quality was inconclusive.

SAP and CS are sensitive measures to assess an observer’s visual function, applicable in both fundamental research and clinical settings. Therefore, the aim of this study was to assess the effect of two commercially available yellow filters, each with a distinct visible light transmission (VLT), on the visual function of normal individuals by using white-on-white perimetry and CS testing.

## Materials and methods

Study participants

This was a comparative study involving 31 eyes of 31 healthy individuals who visited the Ophthalmology Department of the 401 General Military Hospital of Athens, Greece, between May and December 2021. After enrolling about half of our patients and having our first results, we performed a sample size calculation using the G*Power software (Heinrich Heine University Düsseldorf, Düsseldorf, Germany). From this a priori analysis, with an effect size of 0.5, type I error probability of 0.05, and study power of 0.80, we estimate that we need a total sample size of 21 subjects. The study protocol was in accordance with the Declaration of Helsinki and was approved by the Research Ethics Committee of the University of West Attica (approval protocol number: 36659; date: April 27, 2021) and by the Scientific Council of the 401 General Military Hospital of Athens (meeting protocol number: 05; date: June 2, 2020). Informed consent was obtained from all participants.

The inclusion criteria were the following: (a) male or female who are 18-80 years of age, (b) intraocular pressure (IOP) of ≤22 mmHg with no history of increased intraocular pressure (IOP), (c) the absence of glaucomatous disc appearance, (d) a normal visual field (VF) result defined as a mean deviation (MD) and pattern standard deviation (PSD) within 95% confidence limits and a glaucoma hemifield test (GHT) result within normal limits, (e) reliable VF (≤33% false positives, false negatives, and fixation losses), (f) able and willing to make the required study visit, (g) able and willing to give consent, and (h) both eyes meeting the criteria for the control to be included in the study. The exclusion criteria included the following: (a) a history of amblyopia; (b) corrected visual acuity of less than 0.2 logarithm of the minimum angle of resolution (logMAR) (20/30) in either eye if age 50 or older and less than 0.1 logMAR (20/25) in either eye if under age 50; (c) refractive error in either eye exceeding 5 diopter (D) spherical equivalent or 1.0 D cylinder; (d) suspicious or pathological optic discs; (e) VF defect or a suspicion of a VF defect in the tested eye that was explained by ocular status or history; (f) previous or current significant ophthalmic disease in the tested eye, significant eye trauma or intraocular surgery, or the presence of ocular findings that could affect the VF; (g) abnormal pupil or a history of use of pilocarpine or other medication or a history of disease that might have been affecting pupil size or reactivity; (h) any systemic disease or a history of treatment with medications, any of which may be expected to affect the VF; and (i) a history of stroke, insulin-dependent diabetes, or diabetic retinopathy.

During the recruitment visit, besides the current study’s investigation assessment (CS and SAP), each participant underwent a complete ophthalmological examination that included the following: (a) medical history, (b) best-corrected visual acuity (BCVA) with digital Snellen chart, (c) slit-lamp biomicroscopy, (d) IOP measurement with a Goldmann tonometer, and (e) macular and optic nerve head imaging with swept-source optical coherence tomography (SS-OCT) (DRI OCT Triton, Topcon, Tokyo, Japan). All examinations for each healthy participant were performed and completed within a week.

For patients that met all the inclusion criteria, if both eyes were eligible, one eye was randomly selected. All patients underwent the following investigations with the use of the yellow filter Kiros® (KIROS) and the yellow-orange filter Lumior® (LUMIOR): (1) CS examination with the Pelli-Robson (PR) chart (chart 3) and (2) SAP using the Swedish Interactive Thresholding Algorithm (SITA) 24-2 strategy (Humphrey field analyzer {HFA} II 740, Carl Zeiss Meditec, Jena, Germany). To minimize bias on the VF and CS data, we randomized the sequence of the SAP and PR testing with KIROS and with LUMIOR.

Yellow filters

Essilor’s filters Kiros® and Lumior® both absorb short wavelengths (below 400 nm). According to the manufacturing company, these filters offer maximum protection from ultraviolet (UV) radiation, improving contrast sensitivity, contributing to visual comfort, and in some cases improving visual acuity [20]. Additionally, these are intended for people with vision problems, without however citing any studies on which these assumptions were based [21].

The yellow filter Kiros 1-400

**Figure 1:**
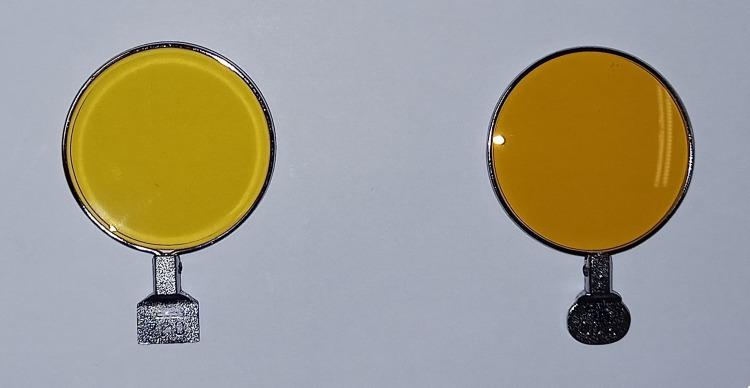
Yellow filter Kiros (left) and yellow-orange filter Lumior (right)

The filter Kiros 1-400 (Kiros_1) has a VLT of 75%, belongs to category 1 (limited protection from sun glare, VLT of 43%-80%) [22], and is light yellow in color. It blocks the transmission of blue light up to 400 nm and specifically transmits wavelengths close to the average maximum sensitivity of the eye (555 nm) (Figure 1 and Table 1) [23,24].

**Table 1:** The special characteristics of filters Kiros® and Lumior® UV, ultraviolet; VLT, visible light transmission

	Kiros	Lumior
Material	Spherical plastic single-vision Orma UVX	Spherical plastic single-vision Orma UVX
Index	1,502	1,502
Abbe number	58	58
Density	1.32	1.32
UV cutoff	400 nm	400 nm
UVA	100%	100%
UVB	100%	100%
VLT	75%	65%
50% VLT	475 nm	515 nm
Tint	Yellow	Yellow-orange
Coating	Antireflective	Antireflective

The yellow-orange filter Lumior 1-400

The yellow-orange filter Lumior 1-400 (Lumior_1) has a VLT of 65%, belongs also to category 1, and has a dark yellow-orange color. It transmits specifically in the central zone of the visible spectrum and filters all ultraviolet and blue radiation up to 400 nm (Figure 1 and Table 1).

**Figure 2:**
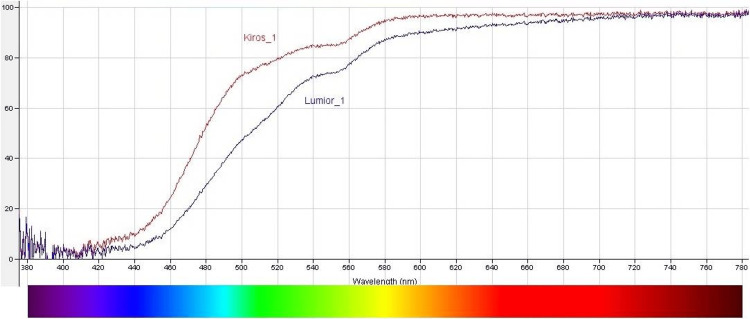
Transmission curves for filters Yellow filter Kiros 1-400 nm (Kiros_1) and yellow-orange filter Lumior 1-400 nm (Lumior_1). Performed at the University of West Attica, Optics and Optometry Department, Metrology Laboratory

Before the beginning of the study, the two lenses were tested for transmission characteristics in the Metrology Laboratory of the Optics and Optometry Department at the University of West Attica, Athens, Greece (Figure 2).

The two yellow filters were cut on an automatic ophthalmic lens wheel and placed in the special metal housing of the ophthalmic lenses used to perform the test on the VF on the Humphrey field analyzer. The lenses within the metal housing were also suitable to be mounted on a test frame for contrast sensitivity assessment purposes (Figure 1). The differences between the two filters are the VLT and their color.

Standard automated perimetry

SAP was performed with the Humphrey field analyzer model II 740 (Carl Zeiss Meditec, Jena, Germany). The strategy chosen was SITA 24-2, stimulus size III (4 mm^2^) with background luminance intensity in apostilbs (31.5 asb).

White-on-white SAP measures the differential light sensitivity at various predetermined retina locations. This is achieved through automated static perimetry, and the following parameters are evaluated: the glaucoma hemifield test (GHT) in categorical classification, the mean deviation (MD) in dB, pattern standard deviation (PSD) in dB, and visual field index (VFI) in percentage.

Since all participants lacked prior experience with VF testing, they underwent a “training session” before the actual test. This involved a SITA fast session on both eyes conducted on an earlier date, without the use of any filters. The VF measurements for the study were subsequently carried out twice: once with the Kiros_1 (yellow) filter and once with the Lumior_1 (yellow-orange) filter.

**Figure 3:**
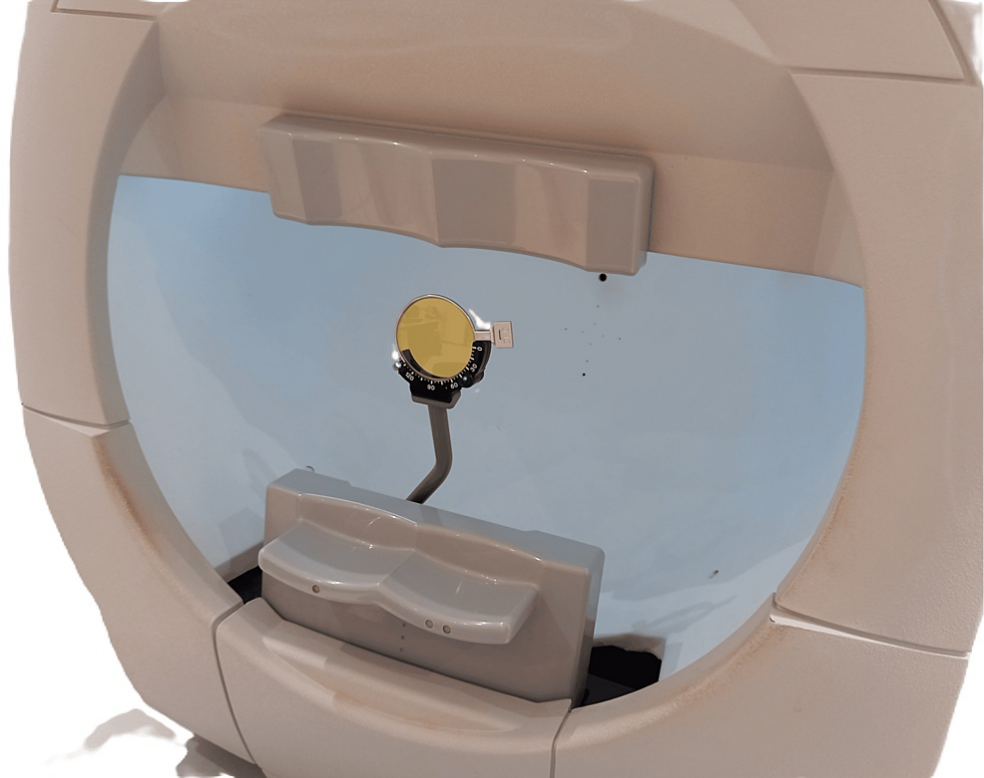
The filter in the metal housing, placed in the cylinder position

The two yellow filters were employed for VF testing in a typical manner, as if using prescription lenses (Figure 3).

Contrast sensitivity

**Figure 4:**
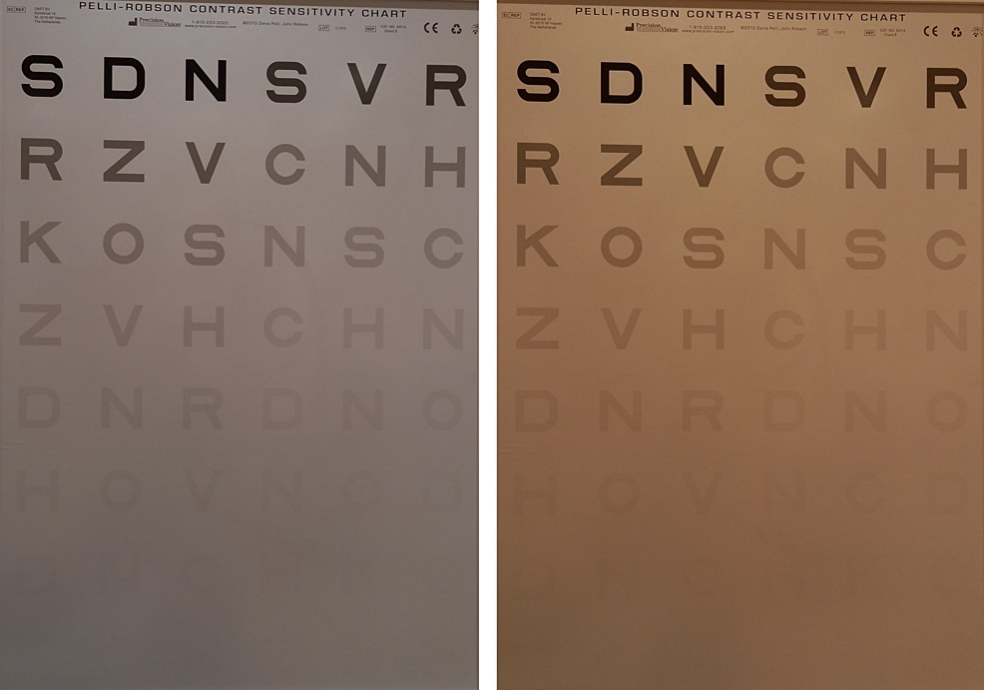
Pelli-Robson chart (chart 3) No filter (left) and with filter Kiros 1-400 (right)

To measure CS, we utilized the uniformly illuminated PR wall chart [25] (chart 3) with a brightness level set at 92.6 cd/m^2^, as illustrated in Figure 4. Individuals were tested at a distance of 1 m and seated with eye height aligned with the middle of the diagram. The best distance correction was applied, or if necessary, an additional 0.75 dpt was added. The chart features large Sloan letters grouped into triplets, with each triplet reduced in contrast by 0.15 log units. The considered contrast ranged from 100% to 0.56%, corresponding to a log from 0.00 to 2.25, respectively. Examinees were deemed to have passed the contrast level if they correctly identified at least two of the three letters in a triplet. In our study, we employed a letter-by-letter scoring system. Each correctly identified letter was assigned 0.05 points. The test concluded when the examinee failed to identify at least two of the three letters in a triplet.

Statistical analysis

Quantitative variables with a normal distribution were analyzed using analysis of variance (ANOVA) for repeated measures and then with the paired-samples t-test for analytic control between pairs. In the non-normal distribution of the quantitative, ordinal, and discrete variables, the Friedman test and the Wilcoxon test were used. Variables with normal distribution were presented as mean value and standard deviation (SD).

Testing for nominal variables was performed using the Cochrane Q test, and these are presented with median value (and interquartile range). For all tests, due to the multiplicity of tests performed, we chose a value of p<0.01 instead of p<0.05 as the level of statistical significance [26].

Distribution testing was done with the Shapiro-Wilk test and histograms. All statistical analyses were performed using the Statistical Package for Social Sciences (SPSS) version 21 (IBM SPSS Statistics, Armonk, NY).

## Results

Thirty-seven consecutive study candidates attended the recruitment visit. Two candidates were excluded due to recent refractive surgery (LASIK), one due to pathological findings in the retinal nerve fiber layer (RNFL) and ganglion cell layer (GCL) performed by optical coherence tomography (OCT), and three due to insufficient reliability in SAP VFs. A total of 31 eyes were included in this study. The demographic and clinical characteristics of the healthy individuals are shown in Table 2.

**Table 2:** Demographic and clinical characteristics of the study’s participants D, diopter; SD, standard deviation; BCVA, best-corrected visual acuity; logMAR, logarithm of the minimum angle of resolution

Characteristic	Results
Age/years (mean {SD})	32.13 (8.13)
Gender (male/female)	14/17
Eye (right/left)	19/12
Sphere (D {SD})	-0.39 (0.85)
Cylinder (D {SD})	-0.20 (0.34)
Spherical equivalent (D {SD})	-0.45 (0.85)
BCVA (logMAR, feet)	1.00, 20/20

Visual fields

The distribution of VF indices for all three study groups is shown in Table 3.

**Table 3:** Descriptive statistics of VF indices VF, visual field; MD, mean deviation; PSD, pattern standard deviation; VFI, visual field index; NF, no filter; KIROS, yellow filter Kiros® 1-400 nm; LUMIOR, yellow-orange filter Lumior® 1-400 nm

Index		Mean (dB)	SD	Median	Minimum	Maximum
MD	NF	-0.59	0.91	-	-	-
	KIROS	-0.56	0.65	-	-	-
	LUMIOR	-0.64	0.71	-	-	-
PSD	NF	1.44	0.21	-	-	-
	KIROS	1.50	0.25	-	-	-
	LUMIOR	1.53	0.23	-	-	-
VFI	NF	-	-	99	97	100
	KIROS	-	-	99	97	100
	LUMIOR	-	-	99	97	100

The VF indices were compared between the three study groups with ANOVA repeated measures, as well as both in pairs (paired-samples t-test). The results are presented in Table 4. No statistically significant difference was detected by the analysis, and all three groups performed similarly in VF testing.

**Table 4:** Comparison results among the three pairs in VF per index VF, visual field; MD, mean deviation; PSD, pattern standard deviation; VFI, visual field index; NF, no filter; KIROS, yellow filter Kiros® 1-400 nm; LUMIOR, yellow-orange filter Lumior® 1-400 nm; ANOVA, analysis of variance

Pairs compared		ANOVA repeated measures (F, p)	Friedman test p-value
MD	NF-KIROS	0.24, 0.788	-
	NF-LUMIOR		
	KIROS-LUMIOR		
PSD	NF-KIROS	1.55, 0.220	-
	NF-LUMIOR		
	KIROS-LUMIOR		
VFI	NF-KIROS	-	0.532
	NF-LUMIOR		
	KIROS-LUMIOR		

In addition, the GHT index (categorical variable) did not show any change with the use of filters compared to the no filter (NF) group, as in all performed VF tests, an indication, “Within Normal Limits,” was shown.

Contrast sensitivity

The mean values, the standard deviation (SD), and the results of the pairwise comparison of the variables of CS (NF, KIROS, and LUMIOR) are shown in Table 5 and Table 6. Pairwise comparisons by ANOVA and t-test revealed significant differences in CS scores among the different filter pairs (Table 6). More specifically, the NF-KIROS pair demonstrated the highest difference in CS scores, followed by NF-LUMIOR. Although the difference between KIROS and LUMIOR was relatively smaller, it remained statistically significant.

**Table 5:** Descriptive statistics of contrast sensitivity indices NF, no filter; KIROS, yellow filter Kiros® 1-400 nm; LUMIOR, yellow-orange filter Lumior® 1-400 nm; SD, standard deviation

Contrast sensitivity	Mean	SD
NF	1.78	0.07
KIROS	1.81	0.06
LUMIOR	1.80	0.06

**Table 6:** Pairwise comparisons between various filter groups CS, contrast sensitivity; NF, no filter; KIROS, yellow filter Kiros® 1-400 nm; LUMIOR, yellow-orange filter Lumior® 1-400 nm; ANOVA, analysis of variance

Pairs compared		Difference	Repeated measure ANOVA (F, p)	Paired t-test p-value
CS	NF-KIROS	0.03	23.08, <0.001	<0.001
	NF-LUMIOR	0.02		<0.001
	KIROS-LUMIOR	0.01		0.003

## Discussion

Our results suggest that the application of Kiros® (yellow) and Lumior® (yellow-orange) filters in healthy individuals had minimal to no effect on SAP results, as they demonstrated statistical equivalence. The use of the Kiros lens resulted in a modest, statistically insignificant reduction in MD by 0.03 dB and a similarly small, nonsignificant increase in PSD from 1.44 dB to 1.50 dB. Conversely, employing the Lumior® lens had a minor negative impact on both MD and PSD indices, with MD increasing and PSD decreasing; however, these changes did not reach statistical significance. The GHT and VFI indices remained unchanged and were unaffected by both yellow filters. Furthermore, when comparing results between the two filters, no significant differences were observed.

The absence of substantial changes in the VF global indices, both statistically and clinically, could be attributed to two factors. Firstly, both filters exhibit only a marginal reduction in the transmission of the visible spectrum (yellow, 75%; yellow-orange, 65%). Secondly, the wavelength spectrum of both lenses, particularly below 400 nm, did not have an impact on the light stimulus used in SAP.

The results of our study are in agreement with the work of Castro et al. [14], who evaluated the influence of a blue-light spectrum filter (BPI® filter number 37870, 450 nm, very bright lemon-yellow filter) on SAP, in healthy individuals. In their study, the filter was designed to closely match the light transmittance level with the spectral transmittance curve of the AcrySof Natural intraocular lens (IOL) (Alcon Laboratories, Inc., Fort Worth, TX), with a cutoff at 400 nm [14,26]. Similar to our results, the authors noted an absence of significant changes in MD and foveal threshold when using a blue-light spectrum filter in healthy individuals.

GHT and VFI indices remained unaltered. The GHT index showed no change, possibly reflecting the fact that the decrease in light transmission caused by the yellow filters was uniform and widespread across the entire VF. The VFI quantifies the extent of visual field loss as a percentage compared to the sensitivity of a reference group of healthy individuals and places a greater weight on locations in the paracentral VF [27]. In our study, VFI values were also not altered by the use of filters, thus confirming the close relationship between MD and VFI [28].

In terms of CS, our results demonstrated a statistically significant improvement (p<0.01), when either of the two filters was used. However, this may not necessarily be considered clinically significant, as an average improvement of around 0.03 log CS corresponds to less than a one-letter difference. These findings indicate that both tested filters performed similarly well, aligning partially with the company’s instructions for use under specific conditions when contrast sensitivity is affected. The two “contrast-enhancing filters” absorb diffuse UV and blue radiation while transmitting specific wavelengths close to the eye’s maximum sensitivity level (average wavelength at 555 nm) [20,23,24].

In contrast to our study’s findings, a previous study in the same field presented conflicting results. Slica et al. [4] used the PR chart to evaluate the effect of the yellow and grey filters on CS in healthy individuals under similar illumination conditions. Measurements were carried out binocularly, encompassing both photopic (background luminance: 60 cd/m^2^) and mesopic conditions (0.35 cd/m^2^). The authors found no significant enhancement in contrast sensitivity with the use of yellow filters. Several factors may account for the divergent outcomes observed in our study favoring yellow filters. Firstly, there were shared characteristics between the filters used in our study and those in Slica et al.’s [4], including refractive index (1.502 versus 1,498), yellow color, Abbe number (58 versus 59.3), density (1.32 versus 1.31), and visible light transmittance (VLT) (75% and 65% versus 67%). However, differences existed in wavelength spectral transmittance (400 versus 475 nm) and coating (antireflective versus reflective). In addition, we evaluated CS monocularly, while Slica et al. [4] examined their subjects binocularly. There was also a difference between the two studies in the luminance of the utilized chart (92.3 versus 60 cd/m^2^). Furthermore, in our work, the analysis of the results was letter-by-letter counting (0.05) instead of triplet counting (0.15).

Based on the findings of our study, we assume that these yellow filters could be considered for use by glaucoma patients without adversely affecting their VF performance. Moreover, since CS is known to be impacted in glaucoma patients [29] and our study demonstrated some improvement in CS with these yellow filters, we may recommend their use as a beneficial aid for individuals with glaucoma. To further support our hypothesis, an ongoing study conducted by our team is specifically focused on assessing the impact of these yellow filters on glaucoma patients. Additionally, we propose that these filters might be recommended for postoperative use by individuals undergoing cataract surgery with premium multifocal intraocular lenses (IOLs). This application could potentially enhance contrast sensitivity, which is known to be affected by certain multifocal IOLs [30,31].

Our study has several limitations. CS was assessed using the Pelli-Robson chart method, which exclusively measures contrast sensitivity at one low spatial frequency (0.5-1 cycle per degree) [32]. This method does not include measurements at intermediate and higher spatial frequencies, aspects not considered in our study. The Pelli-Robson chart’s emphasis on low spatial frequencies, while relevant for evaluating functional vision in everyday tasks such as reading, recognizing faces, and navigating the environment, has a limitation. It does not encompass sensitivity across a broader spatial frequency range, thus restricting the comprehensive evaluation of contrast sensitivity. Additionally, our study is constrained by a relatively small sample size, which diminishes the statistical power and increases the risk of type 2 errors.

The evaluated yellow filters are commercially available. We propose similar studies to be carried out with filters of similar characteristics in healthy controls, as well as in patients with various ophthalmological or neurological diseases. Such studies should include the assessment of visual performance with both CS and VFs.

## Conclusions

In conclusion, we have demonstrated that the use of the yellow filters, yellow Kiros® 1-400 and yellow-orange Lumior® 1-400 in healthy individuals, significantly improves CS in photopic conditions, without affecting their VF performance. However, to generalize these results and evaluate the potential benefits in pathological eye conditions such as glaucoma and age-related maculopathies, further studies involving larger sample sizes are required.
